# Characterizing *PTP4A3*/PRL-3 as the Potential Prognostic Marker Gene for Liver Hepatocellular Carcinoma

**DOI:** 10.1155/2022/2717056

**Published:** 2022-09-30

**Authors:** Xin Jin, Haida Shi, Zhi Li, Huixing Li, Huanxian Ma, Xianjie Shi

**Affiliations:** ^1^Medical School of Chinese PLA and Faculty of Hepato-Biliary-Pancreatic Surgery, The First Medical Center of PLA General Hospital, Beijing 100853, China; ^2^Emergency Department of the Fourth Medical Center of PLA General Hospital, Beijing 100853, China; ^3^Department of General Surgery, General Hospital of Eastern Theater Command, Nanjing 210002, China; ^4^Department of Hepatobiliary Surgery, Aerospace Center Hospital, Beijing 100049, China; ^5^Department of Critical Care Medicine, Faculty of Hepato-Biliary-Pancreatic Surgery, The First Medical Center of PLA General Hospital, Beijing 100853, China; ^6^Medical School of Chinese PLA and Department of Hepatobiliary and Pancreatic Surgery, The Eighth Affiliated Hospital of Sun Yat-Sen University, Shenzhen 518033, China

## Abstract

**Background:**

A large number of cancer-related deaths in the world can be attributed to liver hepatocellular carcinoma (LIHC). The purpose of this study is to explore protein tyrosine phosphatase type IV A member 3 (*PTP4A3*/PRL-3) as a new and reliable biomarker to predict the prognosis of LIHC and determine the potential therapeutic targets or drugs that can be used for treating LIHC.

**Methods:**

We included three LIHC datasets with clinical information and expression profiles from public databases. The expression level of *PTP4A3* was analyzed, and based on the results, the samples were divided into high- and low-expression groups. The Kaplan–Meier survival analysis method was used to determine the relationship between *PTP4A3* and prognosis. The enrichment differences among the functional pathways associated with the high- and low-expression groups were determined using the gene set enrichment analysis (GSEA) method. Five methods were used to determine the differences among the tumor microenvironment in the low- and high-expression groups. The sensitivity of the low- and high-expression groups toward different drug treatment methods was predicted by analyzing the Tumor Immune Dysfunction and Exclusion (TIDE) scores and determining the biochemical half-maximal inhibitory concentration (IC50).

**Results:**

The expression levels of the LIHC and adjacent samples were analyzed, and it was observed that the expression level of *PTP4A3* in tumor tissue was significantly higher than the expression level of the same gene in the adjacent samples. It was also inferred that it might be a cancer-promoting gene. It was concluded that high-expression results in a significantly poor prognosis. The high-expression group was significantly enriched in the tumor-related pathways, such as the PI3K-AKT signaling pathway. In addition, the results obtained by conducting immune infiltration analysis revealed a significant positive correlation between some immune scores and the gene *PTP4A3*. The drug KIN001−135 and gene *PTP4A3* were also found to correlate positively with each other. CP466722, Pyrimethamine, AKT inhibitor VIII, Embelin, Cisplatin, QS11, Bexarotene, and Midostaurin negatively correlated with *PTP4A3* associated with the three datasets. Moreover, the drugs Cisplatin, QS11, Midostaurin, and CP466722 were more sensitive toward the high-expression group than the low *PTP4A3* expression group. Significant differences were observed in these cases.

**Conclusion:**

*PTP4A3*/PRL-3 is potentially associated with the progression, metastasis, and invasion of LIHC. The prognosis of LIHC patients is negatively impacted by the high-expression levels of the gene. The results indicate that *PTP4A3*/PRL-3 is an important prognostic factor for LIHC and is a new potential prognostic detection target. The discovery of the 8 drugs that were negatively associated with *PTP4A3* provided a new direction that can be developed in the future for the treatment of LIHC.

## 1. Introduction

The most common type of primary liver cancer is liver hepatocellular carcinoma (LIHC). The incidence and mortality rate of LIHC hold on a high level especially in ages over than 40 [1] and are closely related to advanced liver disease [2–5]. This malignancy is considered the primary cause of death in patients with liver cirrhosis. Liver cirrhosis is also an important indicator that is used for monitoring and screening the occurrence of LIHC [6–8]. Although immense progress has been made in the field of surgery and medicine, LIHC is one of the most common causes of tumor-related deaths in the world. Most patients suffering from LIHC are diagnosed at an advanced stage of the disease. Patients at an advanced stage of LIHC cannot be treated following the process of surgical resection [5]. The survival rate recorded for most of the patients treated surgically was found to be poor [9–11]. It has been observed that there is a lack of useful prognostic markers for the prognosis prediction of LIHC. Patients who are at a similar tumor stage or are characterized by a similar pathological structure may have a significantly different prognosis, and this can be attributed to individual differences [12, 13]. Therefore, it is important to explore new and reliable biomarkers to predict the prognosis of LIHC.

Apart from the phosphorylation-related enzyme protein tyrosine kinase [14], the phosphorylation-related enzyme protein tyrosine phosphatase (PTP) is a suitable therapeutic target for cancer [15]. It also significantly affects the processes of tumorigenesis and progression [15]. It has been widely reported that PTPs are potential therapeutic targets [16–18]. However, the amount of data available on the role of PTPs in LIHC is lesser than the amount of data available on the role of protein tyrosine kinases (PTKs). Various PTK inhibitors, such as sorafenib and regorafenib, are prescribed to patients in their advanced stages of LIHC [19]. However, high survival rates and satisfactory results have not been obtained using these drugs. PTPs also regulate the process of protein phosphorylation. An imbalance in the levels of PTKs (or PTPs) can result in the abnormal phosphorylation of various downstream proteins. Therefore, we focused on the therapeutic potential of PTPs for the treatment of LIHC.

The protein tyrosine phosphatase 4A (PTP4A) family is commonly known as the phosphatases of regenerating liver (PRL). This family consists of three members of phosphatases and plays an important carcinogenic role in various human cancers. The analysis of literature reports reveals that multiple PRL inhibitors have been reported over the years [20]. Therefore, it is important to understand the role of PRLs in the incidence and progression of LIHC. Previous studies have shown that PRL-1 is overexpressed in LIHC and promotes LIHC cell migration and invasion through endothelial mesenchymal transformation (EMT) [21]. It has also been reported that the prognosis of patients is negatively affected by the upregulation of PRL-3 in LIHC [22, 23]. However, comprehensive information on the molecular mechanism associated with PRL-3 (also known as PTP4A3, hereinafter collectively referred to as PTP4A3) that promotes the development of LIHC is not yet available.

We first analyzed the expression profile and prognosis based on the mRNA expression levels and clinical data corresponding to patients suffering from hepatocellular carcinoma. The relevant data were obtained from the public datasets. We studied the differential genes in the high- and low-expression groups of *PTP4A3* and analyzed the function of the gene. In addition, we also compared and analyzed the immune microenvironment of different *PTP4A3* expression levels in LIHC. The results revealed that some immune scores correlated positively with *PTP4A3*. Finally, we identified 8 drugs that negatively correlated with *PTP4A3*. The results confirm the carcinogenic effect of PRL-3 in LIHC and help identify new targets and treatment methods for LIHC.

## 2. Methods

### 2.1. Data Source and Preprocessing

The TCGA-LIHC dataset (hereinafter referred to as the TCGA dataset) was originally derived from the Cancer Genome Atlas (TCGA) database. It was downloaded from the UCSC Xena data portal. The database contained RNA sequencing (RNA-seq) data for LIHC and normal samples. It also contained clinical information and somatic mutation data.

The TCGA-LIHC dataset (hereinafter referred to as TCGA dataset) was originally derived from The Cancer Genome Atlas (TCGA) database. It was downloaded from the University of California Santa Cruz (UCSC) Xena data portal (https://xenabrowser.net/). The dataset contained RNA sequencing (RNA-seq) data corresponding to LIHC and normal samples. It also contained clinical information and somatic mutation data. For RNA-seq data, the data in the fragments per kilobase of transcript per million mapped reads (FPKM) format were converted to the transcript per million (TPM) format. Following this, log2 conversion was realized. The downloaded somatic mutation data contained information on single nucleotide variations (SNVs) and copy number variations (CNVs). The SNV data were processed using MuTect tool, and the CNV data were processed using the Genomic Identification of Significant Targets in Cancer (GISTIC) algorithm. Data on methylation was downloaded from the LinkedOmics data portal (https://www.linkedomics.org/login.php). Finally, 365 LIHC samples were identified from the TCGA dataset.

The chip data (corresponding to LIHC) were obtained from the Gene Expression Omnibus (GEO) database (https://www.ncbi.nlm.nih.gov/geo/). The GSE14520 dataset was considered, which contained data associated with the expression profile and survival rates. The chip probe was converted into gene symbols. The samples lacking clinical follow-up information, survival time, status, and expression profile data were removed, and 221 LIHC samples were finally included in the studies. The LIHC expression data and survival data for ICGC-LIRI-JP (also known as HCCDB18) were downloaded from the HCCDB website, which was accessed through https://lifeome.net/database/hccdb/download.html. Finally, 203 LIHC samples were included to conduct the studies.

### 2.2. Analysis and Construction of the Survival Model

The TCGA dataset was considered, and the differentially expressed genes (DEGs) in the high- and low-expression groups of *PTP4A3* were identified using the limma package [24]. The genes were filtered based on the threshold false discovery rate (FDR) <0.05 and log2(fold change, FC) >1.5. We analyzed different datasets and used survminer (R-package; accessed through https://CRAN.R-project.org/package=survminer) to obtain the best cut-off values for the genes. The samples were divided into high- and low-expression groups based on the best cut-off values, and the Kaplan–Meier (KM) survival curve was generated.

### 2.3. Gene Set Enrichment Analysis (GSEA) and Functional Annotation

We used “GSEA” [25] for pathway analysis to study the pathways associated with different molecular subtypes. We used the GSEA method using all candidate gene sets in the Kyoto Encyclopedia of Genes and Genomes (KEGG) pathway provided by the MSigDB database to conduct the studies [26]. The input file for GSEA contains the data on the expression profile, and the sample label marks the sample as a high group or a low group. Further, we used the Gene Ontology (GO) function enrichment analysis and KEGG pathway analysis methods to study the differential genes in the LIHC groups using WebGestaltR (v0.4.2; R software) [27]. Subsequently, the results of the “GSEA” pathway analysis were filtered under conditions of *P* < 0.05 and FDR <0.25.

### 2.4. Analysis of Tumor Immune Microenvironment

Convolution and deconvolution are common algorithms in the field of deep learning. Each sample is a mixture of multiple immune cells. The linear regression method is used to fit the relationship between the composition and expression of each immune cell and the final mixture. The expression characteristics of each immune cell are extracted using the deconvolution algorithm. Methods (based on the expression characteristics) to realize the deconvolution of the cell mixtures include the Tumor IMmune Estimation Resource (TIMER2) [28, 29]. TIMER2 is one of the most commonly used immune infiltration analysis methods in the field of bioinformatics analysis. The microenvironment cell populations-counter (MCP-counter; R package) [30] can be used to quantify the absolute abundance of 2 stromal cells and 8 immune cells present in heterogeneous tissues. The package is used to analyze the normalized transcriptome data to arrive at the results. The score presents the degree of infiltration in the immune microenvironment, and the abundance of cells cannot be compared with each other. The Estimation of STromal and Immune cells in MAlignant Tumour tissues using Expression data (ESTIMATE) [31] package cannot be used to score specific immune cell infiltration but can be used to only analyze the purity of the immune cells and tumor cells and the abundance of stromal cells. Estimating the Proportion of Immune and Cancer cells (EPIC) [32] package can be used to analyze the levels of infiltration of the 8 kinds of immune cells (cancer-associated fibroblasts (CAFs), B cells, CD8 + T cells, CD4 + T cells, natural killer (NK) cells, endothelial cells, and macrophages) based on the expression data. The algorithm associated with EPIC uses the constrained least square regression method to explicitly incorporate nonnegative constraints into the deconvolution problem. The algorithm is also used to meet the criterion that the sum of all cell fractions in each sample should be less than one. Under these conditions, based on the operation results, we can directly analyze the differences among various cell components of the sample. For example, a significant decrease in the proportion of B cells, CD8 + T cells, and NK cells indicates that the extent of immune response in the tissue gets inhibited, the recruitment process of immune cells gets hindered, and the tumor immunity gets inhibited. The increasing number of CAFs also contributes the immunosuppressive environment and the development tumors.

Finally, we used EPIC [32], MCP-counter [30], TIMER2 [28, 29], ESTIMATE [31], and ssGSEA [25] to analyze the immune infiltration levels of LIHC and evaluate the tumor immune scores of the samples. In short, EPIC, MCP-counter, TIMER, and ssGSEA can be used to determine the composition of different immune cells. The ESTIMATE package can be used to evaluate tumor purity, stromal cell score, immune cell score, etc., for each sample. The expression of a single gene can be correlated with these immune invasion values. This method considers the marker genes corresponding to different immune cells as the gene set and uses an algorithm similar to GSEA to evaluate whether the highly expressed genes in the sample are enriched in the gene set of different immune cells.

### 2.5. Drug Sensitivity Analysis

The Tumor Immune Dysfunction and Exclusion (TIDE) [33] analysis method can be used to identify biomarkers for the prediction of the efficacy of immune checkpoint inhibitors or drugs by comprehensively analyzing hundreds of different tumor expression profiles. The sensitivity of immune checkpoints can be determined by obtaining the TIDE score following the process of TIDE analysis. In addition, to estimate the risk score of predicting the molecular drug response, we used the “pRRophetic” R software package [34] to evaluate half of the maximum inhibitory concentration (IC50) values of the drugs based on the expression profile obtained from different datasets. Following this, we determined the correlation between these drugs and the gene (*PTP4A3*) in different datasets.

### 2.6. Statistical Analysis

All statistical analysis and visualization methods were performed using R software (4.1.0). Clinical features were expressed as mean ± standard deviation or *n* (%). The Benjamini–Hochberg method was used to control the FDR value. Adjusted *P* values below 0.05 were considered significant. The Pearson correlation analysis method was used to correlate the identified features and clinical parameters.

## 3. Results

### 3.1. *PTP4A3* Expression Is Associated with the Overall Survival

**Figure 1:**
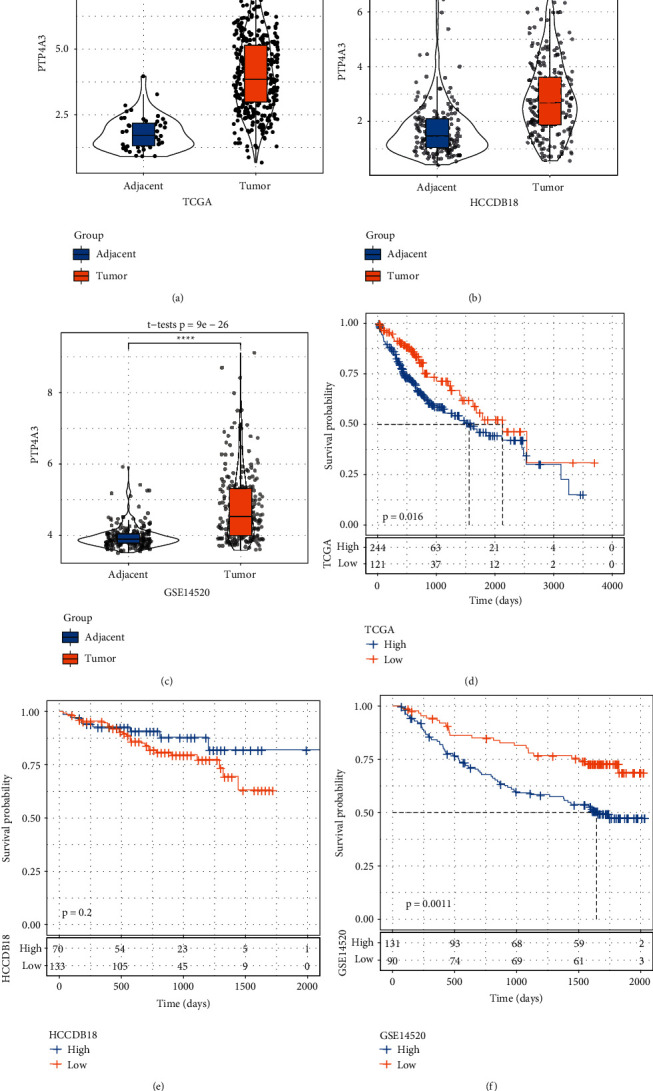
Differences in the PTP4A3 expression levels and survival rates among the different datasets. (a–c) Differences in the expression levels of PTP4A3 for the tumor and adjacent samples of different datasets. (d–f) Survival differences between the low- and high-expression groups for the gene PTP4A3 in different datasets (^*∗*^*P* < 0.05, ^^*∗∗*^^*P* < 0.01, ^^*∗∗∗*^^*P* < 0.001, and ^^*∗∗∗∗*^^*P* < 0.0001).

We first assessed the expression level of *PTP4A3* in tumor and normal (tumor-adjacent) samples. A significantly high differential expression level was observed between the two groups. The *PTP4A3* expression levels of the tumor samples in all three independent datasets (TCGA, HCCDB18, and GSE14520) were significantly high (Figures 1(a)–1(c)), indicating that *PTP4A3* could potentially play an oncogenic role in LIHC. To understand whether the level of *PTP4A3* expression was associated with overall survival, we classified LIHC samples into two groups based on the optimal cut-off value of the *PTP4A3* expression level. The optimal cut-off value was determined following the KM survival analysis method. The results revealed that the two groups (in the three datasets) were characterized by different overall survival rates (Figures 1(d)–1(f)). A significant difference was observed for the TCGA and GSE14520 datasets (*P* = 0.016 and *P* = 0.0011, respectively), while a significant difference was not observed for the HCCDB18 dataset (*P* = 0.2).

There were significant differences between the TCGA and GSE14520 datasets. The groups with high *PTP4A3* expression levels exhibited poor prognoses. LIHC samples were divided into two groups based on the optimal cut-off for *PTP4A3*. The high-expression group of *PTP4A3* was characterized by a higher risk than the low-expression group, indicating that *PTP4A3* was a risk factor for LIHC.

### 3.2. Relationship between the *PTP4A3* Expression Level and Mutation

We further analyzed the samples and found that the mutation frequency of *PTP4A3* in LIHC was 1%. Following this, we mapped the 10 genes that were characterized by the highest mutation frequency in the low- and high-expression groups. The results revealed that the mutation frequencies corresponding to TTN, TP53, CTNNB1, MUC16, ALB, RYR2, and other genes in the high-expression group were higher than the mutation frequencies of the genes belonging to the low-expression group (Figure S1A). It was also observed that *PTP4A3* mutated in the high-expression group but not in the low-expression group (Figure S1B). Subsequently, we analyzed the differences in the TMB associated with the low- and high-expression groups. A significant difference was not observed (Figure S1C).

We also analyzed the amplification and deletion processes associated with *PTP4A3* and compared the expression levels of *PTP4A3* in the different groups. It was found that the group with amplified *PTP4A3* (gain group) was characterized by the highest expression level, which was significantly higher than diploid group (Figure S1D). The correlation between the expression of *PTP4A3* and the degree of methylation was determined and plotted. The results revealed that the expression of *PTP4A3* negatively correlated with the methylation of *PTP4A3* (Figure S1E), suggesting that the higher methylation level corresponded to lower expression level.

### 3.3. DEGs in Different *PTP4A3* Expression Groups

**Figure 2:**
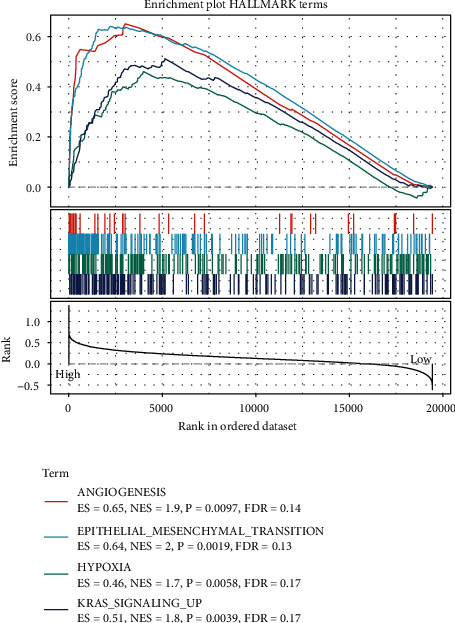
Partial GSEA analysis results obtained for the high and low-expression PTP4A3 groups in the TCGA dataset.

The significantly enriched pathways in the low- and high-expression groups were analyzed using the GSEA method. The results revealed that, in the TCGA dataset, angiogenesis, EMT, hypoxia, and other pathways that were associated with the invasion and metastasis processes were significantly enriched in the high-expression groups. This indicates that the high-expression level of *PTP4A3* can be potentially associated with the processes of metastasis and invasion (Figure 2).

For the TCGA dataset, we obtained 2212 DEGs for the high- and low-expression groups of *PTP4A3*. Of these, 1831 genes were upregulated, and 381 genes were downregulated. For the downregulated genes associated with LIHC, several significant GO function annotation entries were identified (FDR <0.05). Of these, 1093 items with a significant difference in biological process (BP) were annotated (Figure S2A), 158 items with significant differences in cellular component (CC) were annotated (Figure S2B), and 103 items with significant differences in molecular function (MF) were annotated (Figure S2C). For the KEGG pathways enriched by the downregulated expression genes (FDR <0.05), 59 were annotated (Figure S2D). Among them, the ECM−receptor interaction pathways, TNF signaling pathway, pathways associated with cancer, PI3K-AKT signaling pathway, and other tumor-related pathways were significantly enriched.

For the upregulated genes associated with LIHC, several significant GO function annotation entries were identified (FDR <0.05), of which 417 entries with significant differences in BP were annotated (Figure S3A), 30 entries with significant differences in CC were annotated (Figure S3B), and 96 entries with significant differences in MF were annotated (Figure S3C). The upregulated genes associated with LIHC were enriched in the case of the KEGG pathways (FDR <0.05), and 37 entries were annotated (Figure S3D). The pathways associated with retinol metabolism, cholesterol metabolism, metabolism of xenobiotics (in the presence of cytochrome P450), tryptophan metabolism, and other metabolic events were significantly enriched.

### 3.4. Comparative Analysis of the Immune Microenvironment of the Different *PTP4A3* Expression Groups in LIHC

**Figure 3:**
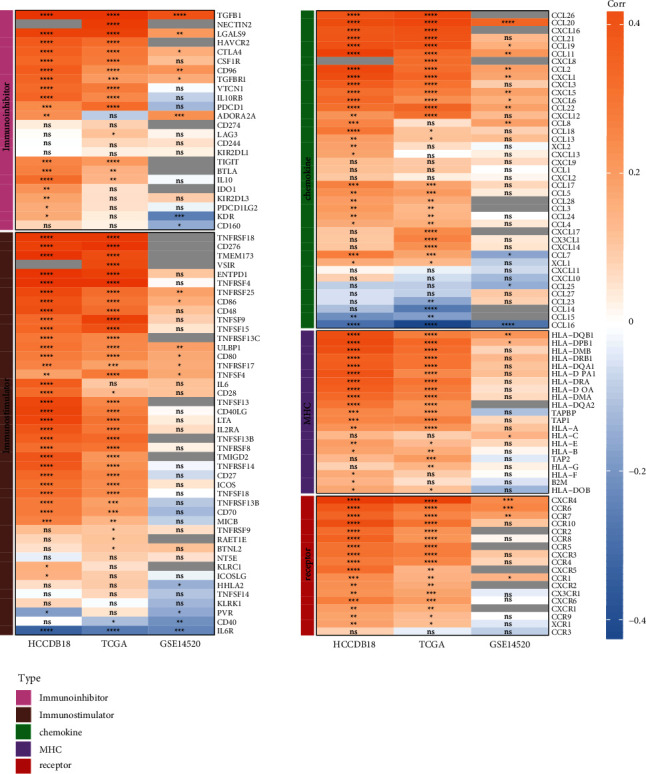
Correlation between PTP4A3 and the five types of immune-related genes in different datasets. ^*∗*^*P* < 0.05, ^*∗∗*^*P* < 0.01, ^*∗∗∗*^*P* < 0.001, ^*∗∗∗∗*^*P* < 0.0001, and ns: *P* > 0.05; Gray indicates that the gene does not exist in the dataset.

We identified 5 types of genes from literature reports [35]. These are associated with chemokine, immunostimulator, immunoinhibitor, major histocompatibility complex (MHC), and receptor. The correlation between *PTP4A3* and these genes was analyzed for different datasets. The results revealed that *PTP4A3* showed a significant positive correlation with these 5 types of genes associated with the TCGA and HCCDB18 datasets. It was also observed that there was no significant correlation between *PTP4A3* and most of these 5 types of genes associated with the GSE14520 dataset (Figure 3).

The differential expression levels of the 5 types of immune-related genes were analyzed by taking into consideration the different expression groups of *PTP4A3* (Figures S4A–S4E). Most of the 5 different types of genes exhibited significant differences, and most of them were highly expressed in the high-expression group.

EPIC, MCP-counter, TIMER, ESTIMATE, and ssGSEA were used to analyze the LIHC immune infiltration levels in different datasets. Following this, the correlation between the *PTP4A3* expression levels and their scores was determined. The results revealed that the *PTP4A3* expression level positively correlated with the immune scores (corresponding to the TCGA and HCCDB18 datasets) determined using different software systems. Most immune scores were not related to *PTP4A3* in the GSE14520 dataset, while some immune scores correlated positively with *PTP4A3*. TIMER was used for the evaluation of CD4 T cells and B cells, and EPIC was used for the evaluation of the CD4 T cells. Three immune scores were determined using ESTIMATE, the MCP-counter software was used for the evaluation of T cells, and the ssGSEA method was used for the evaluation of the activated CD4 T cells, regulatory T cells, NK T cells, activated B cells, etc.

**Figure 4:**
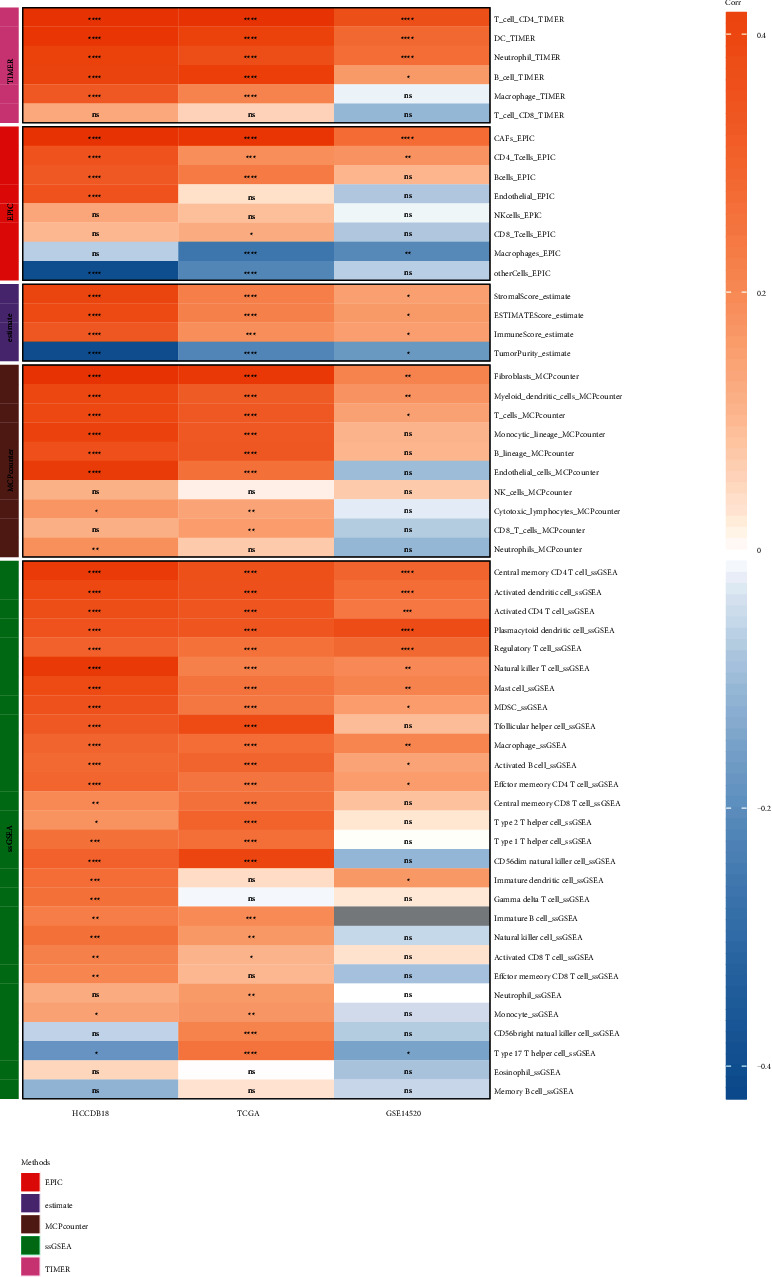
Correlation between PTP4A3 and the five software-based immune scores recorded for the different datasets (^*∗*^*P* < 0.05, ^*∗∗*^*P* < 0.01, ^*∗∗∗*^*P* < 0.001, ^*∗∗∗∗*^*P* < 0.0001, and ns: *P* > 0.05, Gray indicates that the gene does not exist in the dataset).

The differential expression of immune scores obtained using EPIC, MCP-counter, TIMER, ESTIMATE, and ssGSEA for the different expression groups of *PTP4A3* was analyzed (Figures S5A–S5C). We observed that the immune cell enrichment score corresponding to the high-expression group was generally higher than that of the low-expression group. The results agreed well with the results obtained following the correlation analysis method. We continued to analyze the correlation between *PTP4A3* and the 5 software-derived immune scores. Significant correlations between immune scores and *PTP4A3* expression were shown in three datasets, among which is the HCCDB18 dataset (Figure 4).

### 3.5. Relationship between *PTP4A3* and Drug Sensitivity

**Figure 5:**
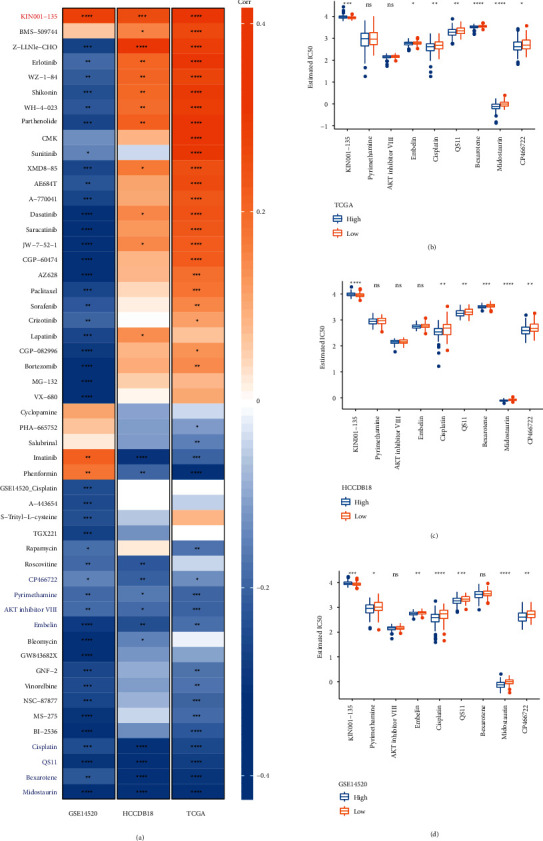
Drug sensitivity analysis of PTP4A3 for the three datasets. (a) Correlation analysis for drug sensitivity and PTP4A3. (b) Distribution of the 9 drugs in the different expression groups of PTP4A3 in the TCGA dataset. (c) Distribution of 9 drugs in different expression groups of PTP4A3 in the HCCDB18 dataset. (d) Distribution of 9 drugs in different expression groups of PTP4A3 in the GSE14520 dataset (^*∗*^*P* < 0.05, ^*∗∗*^*P* < 0.01, ^*∗∗∗*^*P* < 0.001, ^*∗∗∗∗*^*P* < 0.0001, and ns: *P* > 0.05).

The results reveal that the expression of *PTP4A3* correlates significantly with disease prognosis and the tumor microenvironment. This indicated that *PTP4A3* could potentially play an important role in the development of LIHC. Therefore, we believe that *PTP4A3* can be used as a potential drug target. The sensitivity of the low- and high-expression groups toward chemotherapeutic drugs, targeted drugs, and immunotherapeutic drugs was evaluated to validate this hypothesis. The correlation between the 52 drugs and *PTP4A3* for different datasets was determined. It was observed that the 9 drugs correlated significantly with the *PTP4A3* expression levels recorded for the three datasets (Figure 5(a), |R| > 0.15, *P* < 0.05). However, only KIN001−135 positively correlated with *PTP4A3* expression in all three datasets (*P* < 0.0001). The 8 drugs (CP466722, Pyrimethamine, AKT inhibitor VIII, Embelin, Cisplatin, QS11, Bexarotene, and Midostaurin) correlated negatively with *PTP4A3* in the case of all the three datasets.

Next, the differences in the IC50 values of the 9 drugs associated with different *PTP4A3* expression groups in different datasets were compared (student *t*-test, Figures 5(b)–5(d)). The results revealed that the drug KIN001−135 was more sensitive toward the low-expression group of *PTP4A3*(*P* < 0.001). Cisplatin, QS11, midostaurin, and CP466722 were more sensitive toward the high-expression group of *PTP4A3* associated with the three datasets (*P* < 0.01).

**Figure 6:**
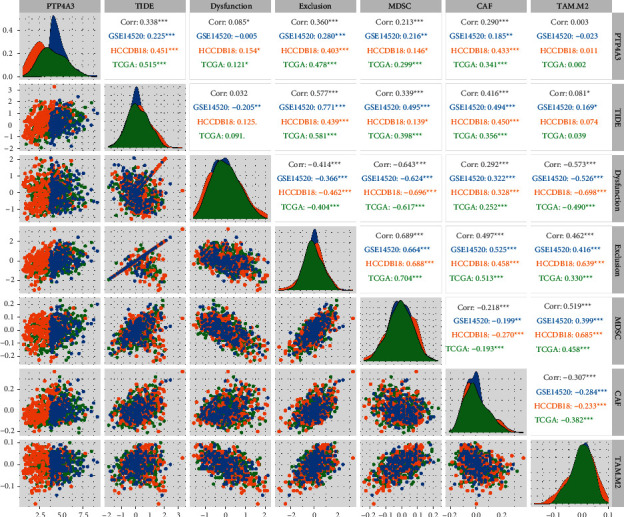
Correlation between TIDE score and PTP4A3 associated with three datasets (^*∗*^*P* < 0.05, ^*∗∗*^*P* < 0.01, ^*∗∗∗*^*P* < 0.001, ^*∗∗∗∗*^*P* < 0.0001, and ns: *P* > 0.05).

The differences in the effects of immunotherapy among the different expression groups of *PTP4A3* belonging to different datasets were analyzed. The potential clinical effects of immunotherapy on the defined groups were assessed using TIDE (https://tide.dfci.harvard.edu/). An increase in the TIDE prediction score resulted in an increase in the possibility of immune escape. This indicated that patients were less likely to benefit from immunotherapy under these conditions. We determined the correlation between *PTP4A3* and TIDE, dysfunction, exclusion, myeloid-derived suppressor cells (MDSSs), CAFs, and tumor-associated macrophage (TAM).M2 scores obtained for different datasets (Figure 6). The results revealed that *PTP4A3* exhibited a significant positive correlation with TIDE, exclusion, MDSC, and CAF when different datasets were studied. This indicates that an increase in the expression level of *PTP4A3* can potentially reduce the positive effects of immunotherapy on patients.

**Figure 7:**
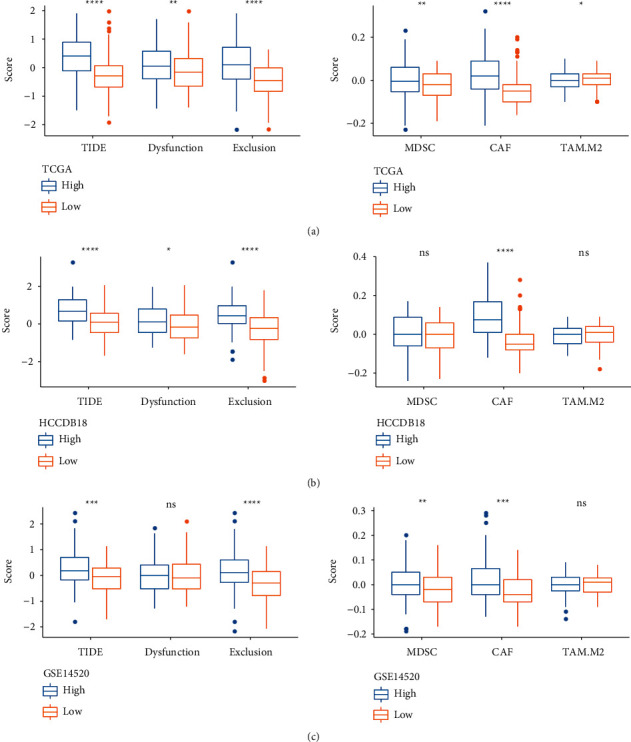
Differences in the TIDE scores associated with three different datasets for different expression groups of PTP4A3. (a) Difference in the TIDE score associated with TCGA. (b) Difference in the TIDE score associated with HCCDB18. (c) Difference in the TIDE score associated with GEO (^*∗*^*P* < 0.05, ^*∗∗*^*P* < 0.01, ^*∗∗∗*^*P* < 0.001, ^*∗∗∗*^*P* < 0.0001, and ns: *P* > 0.05).

We compared the differences in the TIDE scores of different *PTP4A3* expression groups belonging to different datasets (Figures 7(a)–7(c)). Different datasets were studied, and it was observed that the TIDE score corresponding to the high-expression group was significantly higher than the TIDE score recorded for the low-expression group. This indicated that the low-expression group was more responsive toward immunotherapy than the high-expression group. In addition, the exclusion and CAF scores corresponding to the low-expression group were lower than those of the high-expression group.

## 4. Discussion

It has been previously reported that *PTP4A3* plays a variety of roles in the process of cancer metastasis. Cell differentiation, invasion, proliferation, and metastasis are induced by *PTP4A3* when a series of intracellular signaling pathways are activated [36–38]. Moreover, the upregulation of *PTP4A3* in LIHC exerts a negative effect on the patients' prognosis [22, 23]. It also promotes the progress of LIHC [39]. Comprehensive studies on PTKs that function as biomarkers and PTPs that function as tumor markers have not been conducted to identify potential therapeutic targets or drugs that can be used to treat LIHC. Reports in the field are rare, and further studies should be conducted to gain in-depth knowledge. We used three different public datasets for comprehensive expression and drug sensitivity analysis. The results obtained by conducting a comprehensive analysis revealed that *PTP4A3* was a new and reliable potential marker that could predict the prognosis of LIHC. The corresponding targets and drug candidates could also be identified.

We analyzed the TCGA, GEO, and HCCDB18 datasets and observed that the expression levels of *PTP4A3* in tumors were significantly higher than the expression levels recorded for the adjacent samples. This indicated that *PTP4A3* might be a cancer-promoting gene in LIHC. The results agreed well with previously reported results [36]. It was also observed that poor prognosis was associated with high-expression levels of *PTP4A3*. The results revealed that *PTP4A3* dictated patient survival and was associated with poor prognosis. It has been previously reported [22–40] that TGFB1 can be used as a downstream molecule of *PTP4A3*. In other words, the process of TGF-*β* signal transduction mediates the *PTP4A3*-induced FAK activation process. PI3K/AKT and p38 pathways mediate the *PTP4A3*-induced TGFB1 expression and subsequent FAK activation processes, which in turn stimulate the activation of the PI3K/AKT and p38 pathways. This results in the generation of a PRL-3-triggered AKT/p38/TGFB1/FAK positive feedback loop. The results agree well with the results reported herein. The downregulated gene set in the TCGA dataset was significantly enriched in pathways associated with cancer, PI3K-AKT signaling pathway, TNF signaling pathway, and other related pathways. The AKT inhibitor VIII negatively correlated with *PTP4A3* in all three datasets. This was in line with the expectations. The results confirm and validate the reliability of the reported method. The results obtained by conducting GSEA analysis revealed that the high-expression group was significantly enriched in pathways associated with angiogenesis, epithelial mesenchymal transition, hypoxia, invasion, and metastasis, indicating that the high-expression levels of *PTP4A3* could potentially be associated with the processes of metastasis and invasion. The combined results reveal that the influence of *PTP4A3* may result in a poor prognosis, and this can be attributed to the promotion of metastasis and invasion. It has also been reported that *PTP4A3* exhibits significant positive correlation genes associated with chemokine, immunostimulator, immunoinhibitor, MHC, and receptor [35]. The results from immune infiltration analysis revealed that the expression of *PTP4A3* correlated positively with the software-derived immune scores of some of the immune cells. It has been reported by some researchers [38] that *PTP4A3* can upregulate the expression of the chemokine ligand 26 (CCL26) and participate in cell migration. The results from immunohistochemistry (IHC) analysis revealed that the levels of PRL-3 and CCL26 correlated positively with each other, and the levels increased in stages III and IV of colorectal cancer. This could be attributed to the poor prognosis of patients suffering from colorectal cancer. *PTP4A3* can potentially function as a potential prognostic marker in the case of LIHC (similar to the case of colorectal cancer). It promotes the processes of invasion and metastasis associated with LIHC by upregulating CCL26 and inducing the process of TAM infiltration.

The results obtained by analyzing drug sensitivity revealed that CP466722, pyrimethamine, AKT inhibitor VIII, embelin, cisplatin, QS11, bexarotene, and midostaurin correlated negatively with *PTP4A3* associated with the three datasets. AKT inhibition can be considered an attractive therapeutic intervention for LIHC. For example, AKT inhibitor VIII is identified as a drug, which can intervene in LIHC by inhibiting the upstream kinase of AKT signal transduction. The LIHC cell line responds to the AKT inhibitor via the process of apoptotic cell death. The process is independent of the AKT activation state [41]. Among all the drugs, Embelin is an active ingredient that is used in the preparation of traditional herbal medicine. It is used to treat a variety of diseases, such as cancer. It has been reported that Embelin, as a drug delivery system in the liver, is a new candidate drug that can be used for the clinical treatment of advanced hepatocellular carcinoma [42]. Cisplatin can also be used to treat LIHC [43]. These results confirm the reliability of the reported analytical method.

CP466722 has been studied as an inhibitor of ataxia telangiectasia mutated (ATM) kinase [44, 45]. Following the inhibition of ATM, the processes of EMT and tumor metastasis in drug-resistant lung cancer cells are inhibited [44]. In addition, it has also been reported that the ATM inhibitor CP466722 strongly binds to ALK2. A new chemical type for the discovery of drugs for the treatment of progressive ossifying fiber dysplasia was also identified [45]. ALK2 is a serine–threonine kinase receptor (STKR), which belongs to the tyrosine-like kinase (TKL) family. The PI3K-AKT pathway associated with *PTP4A3* is mediated by the processes of serine or threonine phosphorylation associated with a series of downstream substrates. CP466722 has not been previously reported in liver cancer. Therefore, the results reported herein can help provide a platform for the development of drugs that can be used to treat liver cancer.

Further experiments should be conducted to validate the results reported herein. The results reveal whether *PTP4A3* can function as a potential therapeutic target for liver cancer. The impact of the 8 drugs on the processes of tumor progression and the prognosis of patients with liver cancer have also been reported. The effect of immunotherapy on patients suffering from liver cancer needs to be further studied. The results revealed that when different datasets were considered, the TIDE score corresponding to the-high expression group of *PTP4A3* was significantly higher than the TIDE score corresponding to the low-expression group. This indicates that the low-expression group may be more responsive toward immunotherapy. The results from a phase 1 trial underway in Singapore reveal that PRL3-zumab is well tolerated by animals suffering from cancer. The relevant data can be obtained from https://clinicaltrials.gov/ct2/show/NCT03191682?term=PRL-3&rank=1. The safety of using this drug and the preclinical efficacy of PRL3-zumab (studied using an orthotopic tumor model) make the PRL3-zumab-based therapeutic method a safe and effective targeted therapy method.

*PTP4A3*/PRL-3 was identified as one of the molecules that were overexpressed in LIHC tissues. *PTP4A3*/PRL-3 can potentially affect the processes of invasion, progression, and metastasis associated with LIHC. It was observed that the high-expression levels negatively affected the prognosis of LIHC patients. The results indicated that *PTP4A3* should be considered as an important prognostic factor in the case of LIHC. More attention should be paid to the abnormality of *PTP4A3* phosphorylase to gauge the progress of LIHC. It should be considered a potential target for the treatment of LIHC to conduct further research. The results reported herein can potentially help in the development of clinical trials, exploration of treatment methods in preclassified patient groups, and improvement in the survival rate of patients suffering from this fatal disease.

## 5. Conclusion

Overall, the results reveal that the PTP enzyme significantly affects the progression of LIHC. The results from integrated differential expression and correlation analysis revealed that the high-expression levels of *PTP4A3* indicate a poor prognosis. This suggests that the immune scores corresponding to some immune cells correlate positively with the expression of *PTP4A3*. The drug sensitivity analysis method was used to identify 9 potential liver cancer intervention methods and treatment drugs associated with *PTP4A3*. In conclusion, *PTP4A3* should be considered as a potential LIHC prognostic detection target and treatment direction.

## Data Availability

The data used to support the findings of this study are included within the article. The basic data used to support the findings of this study are available from the corresponding author upon request.

## Supplementary Materials

Supplementary Materials Figure S1. Mutation distribution of the low and high PTP4A3 expression groups and the correlation between the expression of PTP4A3 and the methylation level. A: Mutation distribution of the top 10 genes characterized by the highest mutation frequency in the PTP4A3 high-expression group; B: Mutation distribution of the top 10 genes characterized by the highest mutation frequency in the low-expression group of PTP4A3; C: TMB distribution in different expression groups of PTP4A3; D: Expression of PTP4A3 in the PTP4A3 gene amplification group; E: Correlation between PTP4A3 expression and methylation.(^*∗*^*P* < 0.05, ^*∗∗*^*P* < 0.01, ^*∗∗∗*^*P* < 0.001, ^*∗∗∗∗*^*P* < 0.0001 and ns: *P* > 0.05). Figure S2. GO and KEGG annotation of down-regulated DEGs. The top 10 enriched terms were visualized. Figure S3. GO and KEGG annotation of up-regulated DEGs. The top 10 enriched terms were visualized. Figure S4. Expression of five types of immune-related genes in different PTP4A3 expression groups in TCGA-LIHC dataset (A–E: Immunostimulator, chemokine, receptor, MHC, Immunoinhibitor. (^*∗*^*P* < 0.05, ^*∗∗*^*P* < 0.01, ^*∗∗∗*^*P* < 0.001, ^*∗∗∗∗*^*P* < 0.0001, and ns: *P* > 0.05). Figure S5. Distribution of the five software scores for the PTP4A3 groups of the three datasets.
