# Supplementary material for: Characterizing PTP4A3/PRL-3 as the Potential Prognostic Marker Gene for Liver Hepatocellular Carcinoma
Source: J Oncol. 2022 Sep 30;2022:2717056. doi: 10.1155/2022/2717056 (PMC9546693; doi:10.1155/2022/2717056)
Supplement: Supplementary Materials — Figure S1. Mutation distribution of the low and high PTP4A3 expression groups and the correlation between the expression of PTP4A3 and the methylation level. A: Mutation distribution of the top 10 genes characterized by the highest mutation frequency in the PTP4A3 high-expression group; B: Mutation distribution of the top 10 genes characterized by the highest mutation frequency in the low-expression group of PTP4A3; C: TMB distribution in different expression groups of PTP4A3; D: Expression of PTP4A3 in the PTP4A3 gene amplification group; E: Correlation between PTP4A3 expression and methylation.(∗P < 0.05, ∗∗P < 0.01, ∗∗∗P < 0.001, ∗∗∗∗P < 0.0001 and ns: P > 0.05). Figure S2. GO and KEGG annotation of down-regulated DEGs. The top 10 enriched terms were visualized. Figure S3. GO and KEGG annotation of up-regulated DEGs. The top 10 enriched terms were visualized. Figure S4. Expression of five types of immune-related genes in different PTP4A3 expression groups in TCGA-LIHC dataset (A–E: Immunostimulator, chemokine, receptor, MHC, Immunoinhibitor. (∗P < 0.05, ∗∗P < 0.01, ∗∗∗P < 0.001, ∗∗∗∗P < 0.0001, and ns: P > 0.05). Figure S5. Distribution of the five software scores for the PTP4A3 groups of the three datasets. [file 2717056.f1.zip › 2717056.f1/Figure S2.pdf]

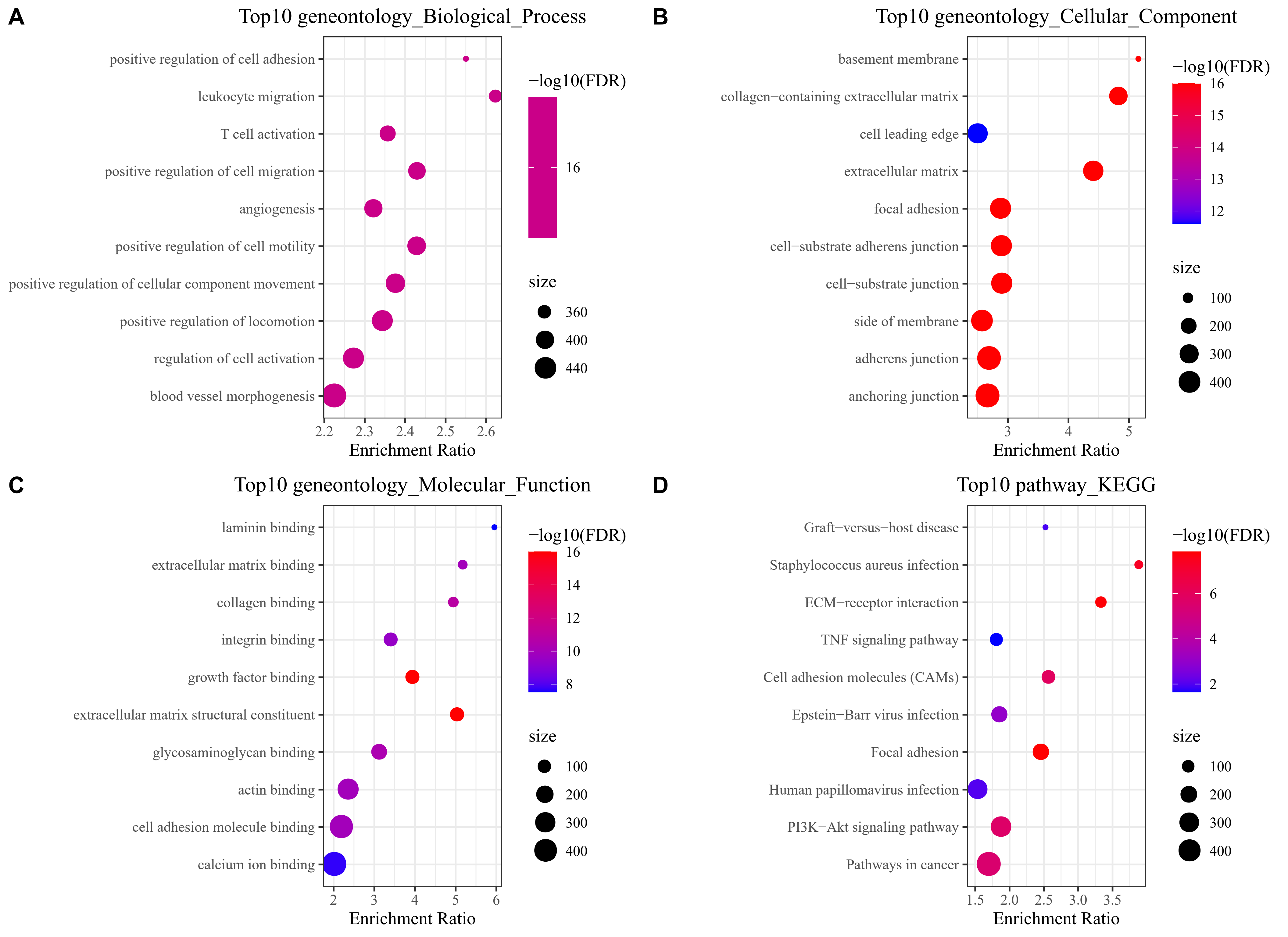
A
Top10 geneontology_Biological_Process
positive regulation of cell adhesion
leukocyte migration
T cell activation
positive regulation of cell migration
angiogenesis
positive regulation of cell motility
positive regulation of cellular component movement
positive regulation of locomotion
regulation of cell activation
blood vessel morphogenesis
2.2 2.3 2.4 2.5 2.6
Enrichment Ratio
−log10(FDR)
16
size
360
400
440
B
Top10 geneontology_Cellular_Component
basement membrane
collagen−containing extracellular matrix
cell leading edge
extracellular matrix
focal adhesion
cell−substrate adherens junction
cell−substrate junction
side of membrane
adherens junction
anchoring junction
3 4 5
Enrichment Ratio
−log10(FDR)
16 15 14 13 12
size
100 200 300 400
C
Top10 geneontology_Molecular_Function
laminin binding
extracellular matrix binding
collagen binding
integrin binding
growth factor binding
extracellular matrix structural constituent
glycosaminoglycan binding
actin binding
cell adhesion molecule binding
calcium ion binding
2 3 4 5 6
Enrichment Ratio
−log10(FDR)
16 14 12 10 8
size
100 200 300 400
D
Top10 pathway_KEGG
Graft−versus−host disease
Staphylococcus aureus infection
ECM−receptor interaction
TNF signaling pathway
Cell adhesion molecules (CAMs)
Epstein−Barr virus infection
Focal adhesion
Human papillomavirus infection
PI3K−Akt signaling pathway
Pathways in cancer
1.5 2.0 2.5 3.0 3.5
Enrichment Ratio
−log10(FDR)
6 4 2
size
100 200 300 400
