# Supplementary material for: Characterizing PTP4A3/PRL-3 as the Potential Prognostic Marker Gene for Liver Hepatocellular Carcinoma
Source: J Oncol. 2022 Sep 30;2022:2717056. doi: 10.1155/2022/2717056 (PMC9546693; doi:10.1155/2022/2717056)
Supplement: Supplementary Materials — Figure S1. Mutation distribution of the low and high PTP4A3 expression groups and the correlation between the expression of PTP4A3 and the methylation level. A: Mutation distribution of the top 10 genes characterized by the highest mutation frequency in the PTP4A3 high-expression group; B: Mutation distribution of the top 10 genes characterized by the highest mutation frequency in the low-expression group of PTP4A3; C: TMB distribution in different expression groups of PTP4A3; D: Expression of PTP4A3 in the PTP4A3 gene amplification group; E: Correlation between PTP4A3 expression and methylation.(∗P < 0.05, ∗∗P < 0.01, ∗∗∗P < 0.001, ∗∗∗∗P < 0.0001 and ns: P > 0.05). Figure S2. GO and KEGG annotation of down-regulated DEGs. The top 10 enriched terms were visualized. Figure S3. GO and KEGG annotation of up-regulated DEGs. The top 10 enriched terms were visualized. Figure S4. Expression of five types of immune-related genes in different PTP4A3 expression groups in TCGA-LIHC dataset (A–E: Immunostimulator, chemokine, receptor, MHC, Immunoinhibitor. (∗P < 0.05, ∗∗P < 0.01, ∗∗∗P < 0.001, ∗∗∗∗P < 0.0001, and ns: P > 0.05). Figure S5. Distribution of the five software scores for the PTP4A3 groups of the three datasets. [file 2717056.f1.zip › 2717056.f1/Figure S3.pdf]

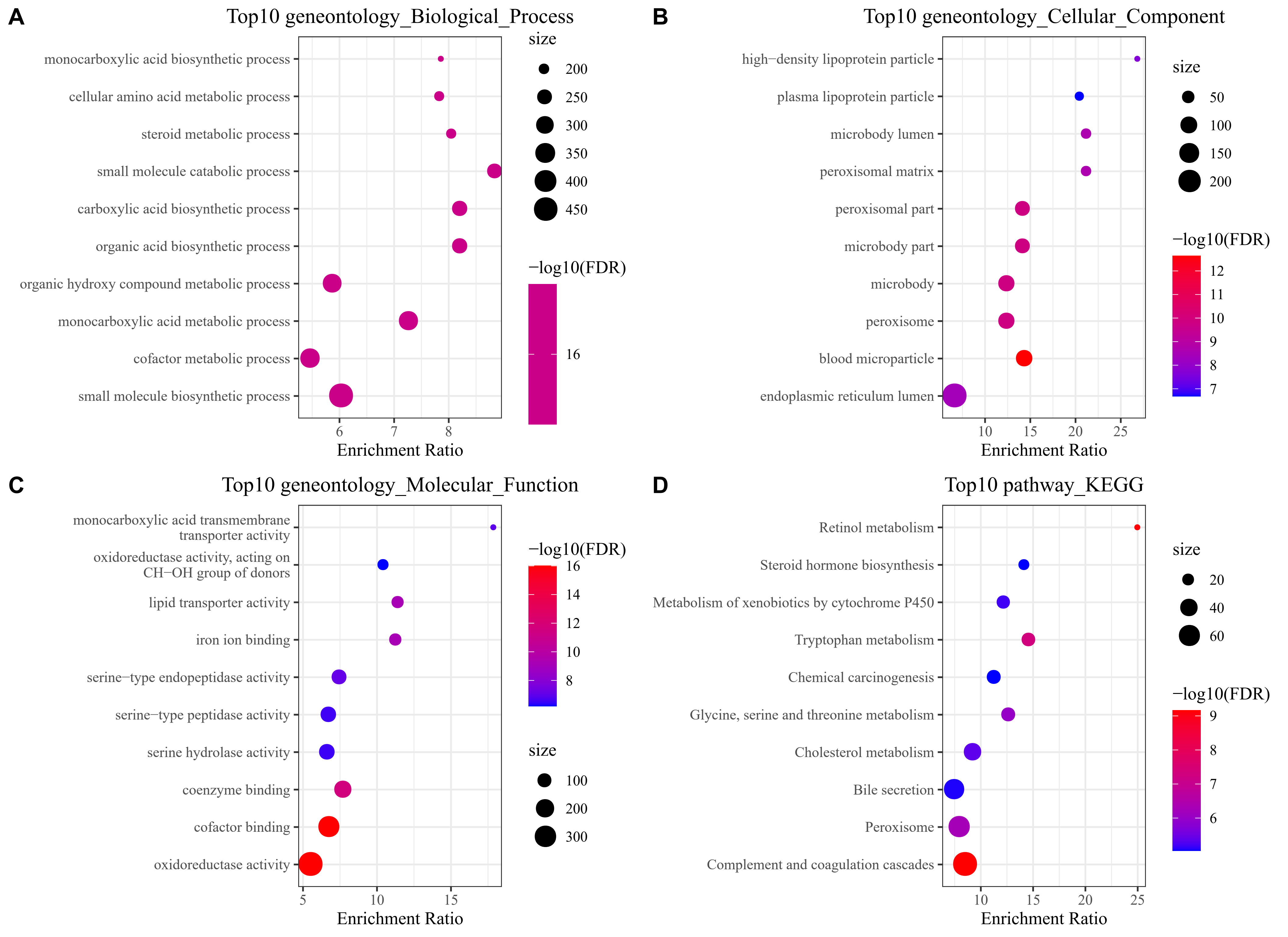
A
Top10 geneontology_Biological_Process
monocarboxylic acid biosynthetic process
cellular amino acid metabolic process
steroid metabolic process
small molecule catabolic process
carboxylic acid biosynthetic process
organic acid biosynthetic process
organic hydroxy compound metabolic process
monocarboxylic acid metabolic process
cofactor metabolic process
small molecule biosynthetic process
6
7
8
Enrichment Ratio
size
200
250
300
350
400
450
−log10(FDR)
16
B
Top10 geneontology_Cellular_Component
high−density lipoprotein particle
plasma lipoprotein particle
microbody lumen
peroxisomal matrix
peroxisomal part
microbody part
microbody
peroxisome
blood microparticle
endoplasmic reticulum lumen
10
15
20
25
Enrichment Ratio
size
50
100
150
200
−log10(FDR)
12
11
10
9
8
7
C
Top10 geneontology_Molecular_Function
monocarboxylic acid transmembrane transporter activity
oxidoreductase activity, acting on CH−OH group of donors
lipid transporter activity
iron ion binding
serine−type endopeptidase activity
serine−type peptidase activity
serine hydrolase activity
coenzyme binding
cofactor binding
oxidoreductase activity
5
10
15
Enrichment Ratio
−log10(FDR)
16
14
12
10
8
size
100
200
300
D
Top10 pathway_KEGG
Retinol metabolism
Steroid hormone biosynthesis
Metabolism of xenobiotics by cytochrome P450
Tryptophan metabolism
Chemical carcinogenesis
Glycine, serine and threonine metabolism
Cholesterol metabolism
Bile secretion
Peroxisome
Complement and coagulation cascades
10
15
20
25
Enrichment Ratio
size
20
40
60
−log10(FDR)
9
8
7
6
