# Supplementary material for: Characterizing PTP4A3/PRL-3 as the Potential Prognostic Marker Gene for Liver Hepatocellular Carcinoma
Source: J Oncol. 2022 Sep 30;2022:2717056. doi: 10.1155/2022/2717056 (PMC9546693; doi:10.1155/2022/2717056)
Supplement: Supplementary Materials — Figure S1. Mutation distribution of the low and high PTP4A3 expression groups and the correlation between the expression of PTP4A3 and the methylation level. A: Mutation distribution of the top 10 genes characterized by the highest mutation frequency in the PTP4A3 high-expression group; B: Mutation distribution of the top 10 genes characterized by the highest mutation frequency in the low-expression group of PTP4A3; C: TMB distribution in different expression groups of PTP4A3; D: Expression of PTP4A3 in the PTP4A3 gene amplification group; E: Correlation between PTP4A3 expression and methylation.(∗P < 0.05, ∗∗P < 0.01, ∗∗∗P < 0.001, ∗∗∗∗P < 0.0001 and ns: P > 0.05). Figure S2. GO and KEGG annotation of down-regulated DEGs. The top 10 enriched terms were visualized. Figure S3. GO and KEGG annotation of up-regulated DEGs. The top 10 enriched terms were visualized. Figure S4. Expression of five types of immune-related genes in different PTP4A3 expression groups in TCGA-LIHC dataset (A–E: Immunostimulator, chemokine, receptor, MHC, Immunoinhibitor. (∗P < 0.05, ∗∗P < 0.01, ∗∗∗P < 0.001, ∗∗∗∗P < 0.0001, and ns: P > 0.05). Figure S5. Distribution of the five software scores for the PTP4A3 groups of the three datasets. [file 2717056.f1.zip › 2717056.f1/Supplementary materials.docx]

Supplementary materials

Figure S1. Mutation distribution of the low and high PTP4A3 expression groups and the correlation between the expression of PTP4A3 and the methylation level. A: Mutation distribution of the top 10 genes characterized by the highest mutation frequency in the PTP4A3 high-expression group; B: Mutation distribution of the top 10 genes characterized by the highest mutation frequency in the low-expression group of PTP4A3; C: TMB distribution in different expression groups of PTP4A3; D: Expression of PTP4A3 in the PTP4A3 gene amplification group; E: Correlation between PTP4A3 expression and methylation.(* P<0.05, ** P<0.01, *** P<0.001, **** P<0.0001, and ns: P>0.05)

Figure S2. GO and KEGG annotation of down-regulated DEGs. The top 10 enriched terms were visualized.

Figure S3. GO and KEGG annotation of up-regulated DEGs. The top 10 enriched terms were visualized.

Figure S4. Expression of five types of immune-related genes in different PTP4A3 expression groups in TCGA-LIHC dataset (A–E: Immunostimulator, chemokine, receptor, MHC, Immunoinhibitor. (* P<0.05, ** P<0.01, *** P<0.001, **** P<0.0001, and ns: P>0.05).

Figure S5. Distribution of the five software scores for the PTP4A3 groups of the three datasets.
